# Colorectal Carcinoma: A General Overview and Future Perspectives in Colorectal Cancer

**DOI:** 10.3390/ijms18010197

**Published:** 2017-01-19

**Authors:** Inés Mármol, Cristina Sánchez-de-Diego, Alberto Pradilla Dieste, Elena Cerrada, María Jesús Rodriguez Yoldi

**Affiliations:** 1 Departamento de Farmacología y Fisiología, Facultad de Veterinaria, Universidad de Zaragoza, 50013 Zaragoza, Spain; ines.marmol9@gmail.com (I.M.); csanchezdg@gmail.com (C.S.-d.-D.); albertopradilladieste@gmail.com (A.P.D.); 2 Departament de Ciencies Fisiologiques II, Universitat de Barcelona, L’hospitalet de Llobregat, 08007 Barcelona, Spain; 3 Departamento de Química Inorgánica, Facultad de Ciencias, Universidad de Zaragoza, 50009 Zaragoza, Spain; ecerrada@unizar.es; 4 Ciber de Fisiopatología de la Obesidad y la Nutrición (CIBERobn), Instituto Agroalimentario de Aragón (IA2), (Instituto de Investigaciones Sanitarias de Aragón IIS Aragó)n, 50008 Zaragoza, Spain

**Keywords:** colorectal carcinoma, ncRNA, microbiota, biomarkers, gene-expression profiling, agarose macrobeads, metal-based drugs, anti-inflammatories, probiotics, functional food

## Abstract

Colorectal cancer (CRC) is the third most common cancer and the fourth most common cause of cancer-related death. Most cases of CRC are detected in Western countries, with its incidence increasing year by year. The probability of suffering from colorectal cancer is about 4%–5% and the risk for developing CRC is associated with personal features or habits such as age, chronic disease history and lifestyle. In this context, the gut microbiota has a relevant role, and dysbiosis situations can induce colonic carcinogenesis through a chronic inflammation mechanism. Some of the bacteria responsible for this multiphase process include *Fusobacterium* spp, *Bacteroides fragilis* and enteropathogenic *Escherichia coli*. CRC is caused by mutations that target oncogenes, tumour suppressor genes and genes related to DNA repair mechanisms. Depending on the origin of the mutation, colorectal carcinomas can be classified as sporadic (70%); inherited (5%) and familial (25%). The pathogenic mechanisms leading to this situation can be included in three types, namely chromosomal instability (CIN), microsatellite instability (MSI) and CpG island methylator phenotype (CIMP). Within these types of CRC, common mutations, chromosomal changes and translocations have been reported to affect important pathways (WNT, MAPK/PI3K, TGF-β, TP53), and mutations; in particular, genes such as c-MYC, *KRAS*, *BRAF*, *PIK3CA*, *PTEN*, *SMAD2* and *SMAD4* can be used as predictive markers for patient outcome. In addition to gene mutations, alterations in ncRNAs, such as lncRNA or miRNA, can also contribute to different steps of the carcinogenesis process and have a predictive value when used as biomarkers. In consequence, different panels of genes and mRNA are being developed to improve prognosis and treatment selection. The choice of first-line treatment in CRC follows a multimodal approach based on tumour-related characteristics and usually comprises surgical resection followed by chemotherapy combined with monoclonal antibodies or proteins against vascular endothelial growth factor (VEGF) and epidermal growth receptor (EGFR). Besides traditional chemotherapy, alternative therapies (such as agarose tumour macrobeads, anti-inflammatory drugs, probiotics, and gold-based drugs) are currently being studied to increase treatment effectiveness and reduce side effects.

## 1. Methodology

**Figure 1:**
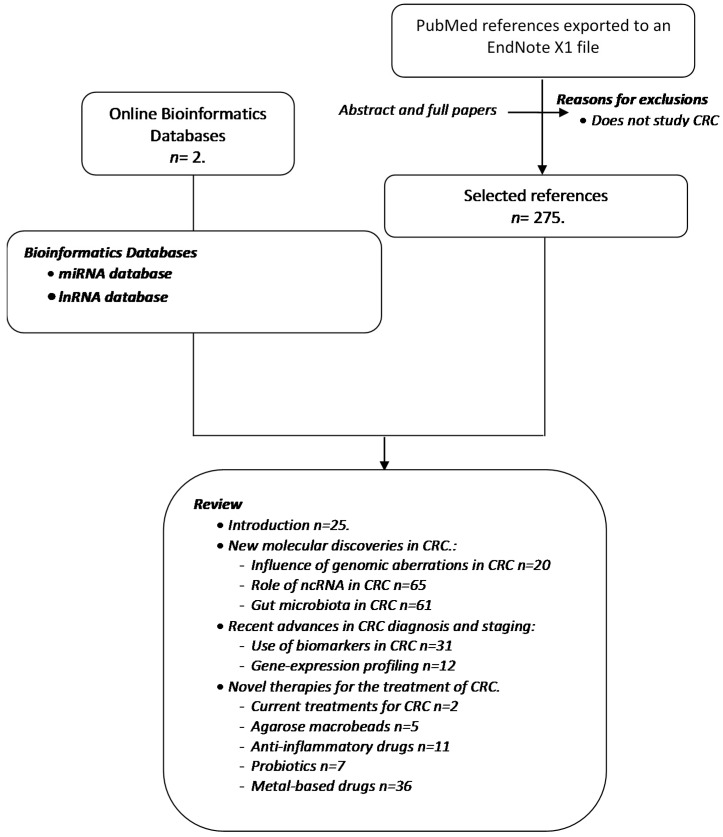
Flowchart displaying the information collection process. Two different data sources were used, namely a search in PubMed and data from online bioinformatics databases. EndNote X1 (Thomson Reuters, New York, NY, USA).

The present report has adhered to systematic review guidelines. All data were collected according to the criteria shown in Figure 1. The search of each of the different parts in PubMed (avaliable on: http://www.ncbi.nlm.nih.gov/pubmed) identified a total of 295 hits from November 1945 to July 2014. In addition, a search was carried out in the databases Long Noncoding RNA Database v2.0 [1,2] and miRCancer: a microRNA-cancer association database [3,4,5] and the results obtained were corroborated by a literature search in PubMed. The combined information from these two sources formed the basis of this review.

## 2. Introduction

### 2.1. Epidemiology

Colorectal cancer (CRC) is one of the most common cancers worldwide, with between one and two million new cases being diagnosed every year, thus making CRC the third most common cancer and the fourth most common cause of cancer-related death, with 700,000 deaths per year, exceeded only by lung, liver and stomach cancers. By gender, CRC is the second most common cancer in women (9.2%) and the third in men (10%) [6]. The incidence of CRC has risen by more than 200,000 new cases per year from 1990 to 2012. Most cases of CRC are detected in Western countries (55%), but this tendency is changing due to the fast development of some countries over the past few years [7]. Even so, only 33% of all CRC-related deaths in the world occur in Western countries in 2010 [8] thanks to the improvements made in health systems and the implementation of screening programs. However, predictions for 2016 are not encouraging at all, with 134,490 new cases of colorectal cancer and 49,190 deaths related to this cancer expected.

### 2.2. Aetiology

Mutations in specific genes can lead to the onset of colorectal cancer, as happens in other types of cancer. Those mutations can appear in oncogenes, tumour suppressor genes and genes related to DNA repair mechanisms [9]. Depending on the origin of the mutation, colorectal carcinomas can be classified as sporadic, inherited and familial.

Point mutations, which appear during life, are not associated with inherited syndromes and only affect individual cells and their descendants. Cancers derived from point mutations are called sporadic cancers, and account for 70% of all colorectal cancers. The molecular pathogenesis of sporadic cancer is heterogeneous as mutations can target different genes [9]. However, approximately 70% of CRC cases follow a specific succession of mutations that is then translated into a specific morphological sequence, starting with the formation of an adenoma and ending in the carcinoma state. The first mutation occurs in adenomatous polyposis coli (*APC*), a tumour suppressor gene, triggering the formation of non-malignant adenomas, also called polyps. Approximately 15% of those adenomas are expected to be promoted to the carcinoma state within a period of ten years. This *APC* mutation is followed by mutations in *KRAS*, *TP53* and, finally, *DCC* [9].

Inherited cancers account for just 5% of all CRC cases. Those cancers are caused by inherited mutations that affect one of the alleles of the mutated gene, meaning that a point mutation in the other allele will trigger the apparition of the tumour cell and, subsequently, the carcinoma. In order to generate a more accurate classification of inherited cancers, two groups, namely polyposis and non-polyposis forms, have been established. The polyposis variant mainly involves familial adenomatous polyposis (FAP), which is characterized by the formation of multiple potentially malignant polyps in the colon [10]. In contrast, hereditary non-polyposis colorectal cancer (HNPCC) is related to mutations in DNA repair mechanisms. The main cause of HNPCC is Lynch syndrome, which is caused by inherited mutations in one of the alleles coding for DNA repair proteins such as MSH2, MLH1, MLH6, PMS1 and PMS2. Lynch syndrome can be found in 2%–3% of all colorectal cancer cases, and is therefore the most common syndrome in the HNPCC group [10,11]. Familial colorectal cancer accounts for approximately 25% of all cases and is also caused by inherited mutations, although they are not classified as inherited cancers per se since they cannot be included in any inherited cancer variant [12].

### 2.3. Risk Factors

Worldwide, the probability of suffering from colorectal cancer is about 4%–5%. Furthermore, many personal traits or habits are considered to be risk factors as they increase the chances of developing polyps or colorectal cancer.

The main risk factor for colorectal cancer is age: past the fifth decade of life, the risk of developing CRC is markedly increased, while the onset of colorectal cancer below the age of fifty is rare (apart from inherited cancers) [13]. In addition to age, there are other inherent risk factors that cannot be modified. A personal history of colorectal cancer or inflammatory bowel disease (IBD)—the risk in patients with ulcerative colitis is increased by 3.7% [14], while people suffering from Crohn’s disease have a 2.5% higher risk of developing colorectal cancer [15]—are also important risks for colorectal cancer development,. The chronic inflammation found in IBD often produces an abnormal cell growth known as dysplasia. Although dysplastic cells are not yet malignant, they have more chances of becoming anaplastic and developing into a tumour. Another risk factor that can be included in this group is the presence of a positive familial history of CRC in relatives, especially those relatives under fifty years of age at diagnosis. An increased risk due to familial history can be derived from inherited mutations or the environment [16].

Some other risk factors, which are related to lifestyle, can be reduced by implementing modest lifestyle changes in terms of dietary and physical activity habits. For instance, it is thought that a sedentary lifestyle can increase the risk of developing colorectal cancer, although this relationship between colorectal cancer and inactivity is not completely defined. However, it has been proved that moderate physical activity increases metabolic rates and gut motility and, in the long term, increases metabolic efficiency and reduces blood pressure [17].

A sedentary lifestyle is also related with obesity, another important risk factor for colorectal cancer. Remarkably, this increased risk is linked to both food intake and increased levels of visceral adipose tissue (VAT), a hormonally active component of total body fat that can promote the development of colorectal cancer through the secretion of proinflammatory cytokines, which leads to an inflammatory situation in the colon and rectum, insulin resistance and modulation of metabolic enzymes such as adiponectin or lectin [18]. In this context, diet is strongly linked to the risk of colorectal cancer such that unhealthy nutritional habits increase the chances of developing colorectal cancer by up to 70% [19]. For instance, red meat releases heme groups in the intestine, which enhance the formation of carcinogenic *N*-nitroso compounds as well as cytotoxic and genotoxic aldehydes by lipoperoxidation [20], and meat cooked at high temperatures produces heterocyclic amines and polycyclic hydrocarbons after digestion, both of which are considered to be potential carcinogens [21]. Furthermore, smoking and alcohol consumption have also been shown to increase CRC risk. In the case of alcohol consumption, acetaldehyde (the main metabolite of ethanol) has been described as carcinogenic by increasing the risk of developing colorectal cancer among populations depending on polymorphisms of alcohol metabolism enzymes [22]. However, the relationship between alcohol consumption and CRC has not yet been totally elucidated. Tobacco smoking, in turn, can increase the chances of suffering from CRC by up to 10.8% [23] due to the high content in carcinogens such as nicotine, the metabolites of which can easily reach the intestine and generate polyps [23,24]. Although smoking increases CRC risk, a significant relationship has only been found for long-term smokers, whether they have stopped smoking or not [25].

### 2.4. Molecular Pathways of Colorectal Cancer

Genomic instability is an important feature underlying colorectal cancer. The pathogenic mechanisms leading to this situation can be included in three different pathways, namely chromosomal instability (CIN), microsatellite instability (MSI) and CpG island methylator phenotype (CIMP).

The CIN pathway, which is also considered to be the classical pathway since it represents the cause of up to 80%–85% of all CRC cases [26], is characterized by imbalances in the number of chromosomes, thus leading to aneuploydic tumours and loss of heterozygosity (LOH). The mechanisms underlying CIN include alterations in chromosome segregation, telomere dysfunction and DNA damage response, which affect critical genes involved in the maintenance of correct cell function, such as *APC*, *KRAS*, *PI3K* and *TP53* amongst others. *APC* mutations cause the translocation of β-catenin to the nucleus and drive the transcription of genes implicated in tumourigenesis and invasion, whereas mutations in *KRAS* and *PI3K* lead to a constant activation of MAP kinase, thus increasing cell proliferation. Finally, loss-of-function mutations in *TP53*, which encodes for p53, the main cell-cycle checkpoint, cause an uncontrolled entry in the cell cycle [27].

The Microsatellite instability pathway is caused by a hypermutable phenotype due to loss of DNA repair mechanisms. The ability to repair short DNA chains or tandem repeats (two to five base-pair repeats) is decreased in tumours with microsatellite instability; therefore, mutations tend to accumulate in those regions. These mutations can affect non-coding regions as well as codifying microsatellites, and tumours develop when reading frames of oncogenes or tumour suppressor genes codified in microsatellites are altered. Loss of expression of mismatch repair genes (MMR) can be caused by spontaneous events (promoter hypermethylation) or germinal mutations such as those found in Lynch syndrome. These tumours are mainly diploid and harbour less LOH. Genes mutated in tumours with microsatellite instability include *MLH1*, *MSH2*, *MSH6*, *PMS1* and *PMS2* [28]. In general, MSI tumours have a better prognosis than sporadic tumours [11].

Epigenetic instability, which is responsible for the CpG island methylator phenotype, is another common feature in CRC. The main characteristic of CIMP tumours is the hypermethylation of oncogene promoters, which leads to genetic silencing and a loss of protein expression. Genetics and epigenetics are not exclusive in colorectal cancer, and both cooperate in its development, with more methylation events than point mutations often being found [29]. One example of the combined effect of genetics and epigenetics in the colorectal cancer development process is the presence of *BRAF* mutations as well as microsatellite instability in many CIMP tumours [30].

## 3. New Molecular Discoveries in Colorectal Cancer (CRC)

### 3.1. Influence of Genomic Aberrations on CRC Outcome

**Figure 2:**
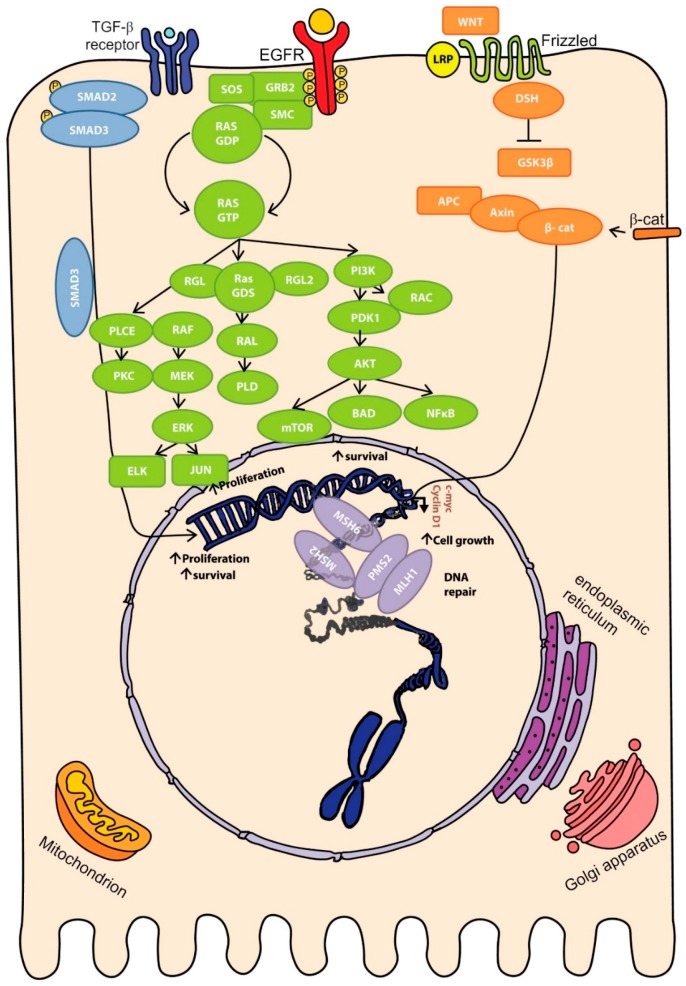
Molecular pathways involved in colorectal carcinogenesis. Mutations affecting proteins involved in WNT (orange), MAPK/PI3K (green), SMAD/TGF-β (blue) or DNA repair (purple) pathways may enhance cell proliferation and survival, thereby inducing tumoural overgrowth and initiating carcinogenesis. Arrow-headed lines indicate protein activation whereas bar-headed lines represent protein inhibition.

New genomic techniques have allowed the identification of a vast number of genomic aberrations involved in colorectal cancer. Thus, although mutations are the main genomic alteration, several chromosomal changes and translocations can also frequently be found in CRC. All of those aberrations affect important pathways (WNT, MAPK/PI3K, TGF-β) and functions within the cell (*TP53* and cell-cycle regulation) [31] (Figure 2).

The WNT pathway plays a key role in stem-cell differentiation and cellular growth. Therefore alterations in this pathway may drive tumour development. WNT pathway alterations in CRC are also associated with weakened tight junctions, which leads to a reduced cellular adhesion and hence favours migration and metastasis [32]. The main genomic aberration in CRC related to the WNT pathway is *APC* mutations, although many other alterations can also target this pathway. Moreover, genomic alterations in the WNT pathway are not exclusive, and tumours harbouring mutations in *APC* may also present other common alterations [31]. Despite being the most common mutated gene, *APC* is not a guaranteed prognostic marker for CRC due to the high frequency of mutations among CRC cases and the wide range of mutations found within this gene [33]. β*-*Catenin, which is also involved in the WNT pathway, is also not generally useful for prognosis since it is commonly overexpressed in CRC cancers [33]. However, overexpression of c-MYC, which is triggered by activation of the WNT pathway, is considered to be a metastasis marker and good survival-related prognostic factor [34,35].

The MAPK and PI3K pathways are both involved in cell proliferation and survival. Alterations affecting these pathways therefore confer proliferative advantages on tumour cells. *KRAS*, *BRAF* and *PIK3CA* (*PI3K*) mutations are the most common type in CRC. Mutations in *KRAS* exon 2 codon 13 are associated with poor prognosis as well as lower survival [36], whereas mutations in exon 2 codon 12 are associated with more advanced tumours and metastasis [37]. In addition to this, *BRAF* mutations also have a poor prognosis associated with lower survival rates, especially in those tumours with microsatellite instability [38,39,40]. Although *BRAF* V600E, which is the most common *BRAF* mutation in many cancer types, is a poor prognostic factor in metastatic cancer [41], it is nevertheless a promising target for personalized medicine, and the combination of specific *BRAF* V600E inhibitors with other MAPK/PI3K pathway inhibitors has been shown to be more effective for treating metastatic CRC [42]. In contrast, *KRAS* and other less common *BRAF* mutations are associated with therapy resistance, hence monotherapy failure leads to poor prognosis [43]. New combinations with MAPK and PI3K pathway inhibitors are needed in tumours with mutated *KRAS* or *BRAF*, which are exclusive. Although mutations in *PIK3CA* are also a common feature in colorectal cancer, the relationship between *PIK3CA* and CRC outcome is not as well established as those involving *KRAS* or *BRAF*. Nevertheless, *PIK3CA* mutations are associated with worse prognosis when accompanied by *KRAS* mutations [44]. Likewise, tumours with combined mutations in exons 9 and 20 of *PIK3CA* have a worse outcome than tumours harbouring just one of those mutations [45]. In addition, loss of *PTEN*, which downregulates the PI3K pathway, in primary tumours is significantly related with an augmented death risk and with poor survival in metastasis [46].

The TGF-β pathway, in turn, plays a role in fundamental cellular processes such as growth, differentiation or apoptosis. However, sporadic mutations in *TGF-β* and its pathway are not particularly common in colorectal cancer, hence they are not significant as prognostic markers [33]. Nevertheless, chromosomal changes involving *TGF-β* are strongly linked to the CIN pathway in CRC. Loss of 18q is one of the main genomic aberrations related to the *TGF-β* pathway in colorectal cancer [47]. Chromosome 18q encodes for two important tumour suppressor genes known as *SMAD2* and *SMAD4*, the loss of which leads to an ability to evade apoptosis and deregulation of the cell cycle. Current studies demonstrate that there is a weak correlation between poor prognosis/shorter survival rates and 18q loss. However, further studies including more extensive cohorts may be needed before 18q loss can be considered to be a helpful prognostic marker [48,49].

Finally, *TP53* is one of the most important tumour suppressor genes and the main cell-cycle checkpoint, thus its loss can drive tumour progression by allowing excessive proliferation. Loss of 17q-*TP53*, which encodes for p53, is a frequent event in CRC as it plays a role in the classical adenoma to carcinoma succession. Moreover, although an association between *TP53* loss and poorer survival rates have been found, *TP53* is not considered a useful prognostic marker since current data are insufficient for its validation [50].

Additionally, overall genomic context of the tumour is of clinical relevance in CRC. The analysis of Dukes’ C CRC found that patients with tumours containing more than two aberrations had a significantly better survival than those with tumours containing two or less aberrations, whereas genomic analysis found no association between specific chromosomal aberrations and survival [51]. Other studies also demonstrated that increased genetic instability [27] or increased chromosomal aberrations are associated with a favourable outcome [51]. The underlying mechanisms are still unclear although it has been suggested that an excess of genomic instability can activate various mechanisms of cell death.

In consequence, molecular characterization of somatic DNA aberrations is a helpful strategy to improve prognosis prediction and therapy selection of individual patients, constituting one of the most important bases for a modern and personalized medicine.

### 3.2. Role of ncRNA in Colon Carcinoma

**Figure 3:**
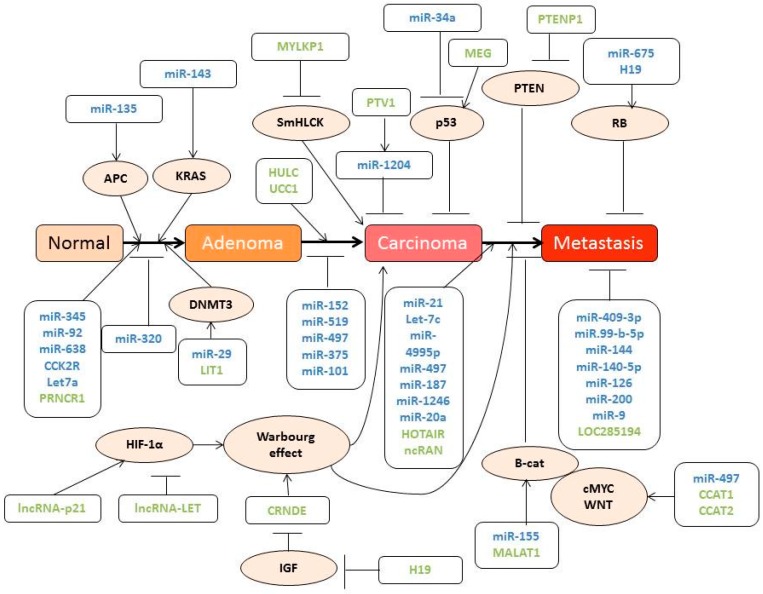
Schematic representation of the different miRNAs (blue) and lncRNAs (green) involved in colorectal carcinogenesis. Arrow-headed lines indicate protein activation whereas bar-headed lines represent protein inhibition.

Non-coding RNAs (ncRNAs) are RNA molecules transcribed from non-coding regions of the genome, thus they lack an open reading frame and are not translated into proteins. In the last few years, several different types of ncRNAs have been identified and associated with several cell functions. For instance, rRNAs and tRNAs participate in mRNA translation, snoRNAs (small nucleolar RNAs) are involved in rRNA modifications, snRNAs (small nuclear RNAs) drive splicing and both miRNAs (micro RNA) and siRNAs (small interfering RNA) regulate gene expression [52]. In this context, miRNAs have been one of the most studied epigenetics elements involved in cancer due to their importance in gene-expression regulation. These miRNAs bind to the 3’UTR regions of several mRNAs, thus inducing their degradation or repressing their translation, consequently associating CRC with deregulation of different miRNAs (Table 1 and Figure 3).

One of the latest discoveries in the field of ncRNAs are known as lncRNAs, which are expressed in many loci of the genome and modulate gene expression in the nucleus and in the cytoplasm. In the nucleus, they can modify epigenetic markers by activating or suppressing chromatin modification proteins such as DNMT3A, whereas in the cytoplasm, they can act as a miRNA decoy, translation regulators or splicing regulators [84]. In consequence, lncRNAs play a critical role in cellular differentiation and proliferation processes, which are intimately involved in cancer.

Over the last few years, and thanks to the development of high-throughput genome sequencing tools, new lncRNAs have been associated with cancer in general, and with CRC in particular. Some of the most important types are presented in Table 2 and Figure 3. Under physiological conditions, these lncRNAs regulate gene expression, epigenetic imprinting or alternative splicing, acting as proto-oncogenes or tumour suppressor genes [85]. When lncRNAs act as proto-oncogenes, their overexpression leads to an upregulation of genes with a critical role in tumour progression, such as *MYC* or genes involved in the WNT signalling pathway [86]. Similarly, when acting as tumour suppressors, lncRNAs regulate the expression of p53-dependent genes under normal conditions. As such, when the expression of these lncRNAs is decreased, the expression of genes under their control becomes altered and cells develop resistance to apoptosis, thereby increasing their proliferation [87]. Moreover, as lncRNA alterations remain constant among patients, they could constitute a potential biomarker for the diagnosis and prognosis of CRC.

The role of ncRNAs can be modified by multiple inherited genetic and epigenetic alterations in addition to structural variations and transcriptional regulations; all of these factors contribute to their dysregulation and are implicated in the etiology and pathophysiology of cancer. It is well established that the dysregulation of ncRNAs alters various cancer-related signalling pathways; however, due to their complicated structural characteristics, as well as their specific temporal and spatial expression patterns; further structural, functional, and mechanistic characterizations should be performed to achieve a better understanding of their particular role in CRC.

Furthermore, a growing number of studies have reported that ncRNAs could function as potential diagnostic and prognostic biomarkers for CRC in stool, serum, plasma, and tissues. As we will develop in the biomarkers section, the most commonly used screening methods for CRC such as colonoscopy and the fecal occult blood test are limited by their invasiveness, low specificity and sensitivity and high cost. Therefore, ncRNAs, thanks to their tissue-specific signature and their particular expression pattern in tumour, have emerged as a great promise for the development of accurate and non-invasive biomarkers for early CRC detection and prognosis prediction. For instance, certain 12 miRNAs (such as miR-7, -17, -20a, -21, -92a, -96, -106a, -134, -183, -196a, -199a-3p, and -214) showed higher expression levels in stool samples from CRC patients than in those from healthy controls, whereas eight miRNAs (miR-9, -29b, -127-5p, -138, -143, -146a, -222, and -938) were shown to be downregulated [117]. Circulating miRNA, can also constitute important biomarkers for CRC screening and prognostic evaluation. Alterations in blood-based miRNAs has been also detected in CRC patients, and they appear to be more sensitive but less specific than fecal miRNAs for the diagnosis of CRC.

However, although several studies have demonstrated the association between certain ncRNA and clinicopathological characteristics, several limitations prevent their use in the clinic. First, the levels of circulating ncRNA transcripts and their post-transcriptional modifications are unstable and variable or even difficult to detect during different stages of the disease. Second, there is not a simple standard assay or consensus in an endogenous control for the quantification of circulating ncRNAs. Third, most of the obtained results have not been replicated and, moreover, ncRNA-based diagnoses appear to be more accurate in Asian than in Caucasian patients [118]. In consequence, the actual results need to be confirmed in multiple studies with larger-scale validation across multiple centers and different populations. Finally, it is difficult to determine the origins of circulating ncRNAs that have been isolated and quantified, and hence it is hard to determine whether these ncRNAs are secreted from cells of tumour tissues or whether they are detected as a consequence of hematocyte contamination.

Besides their potential use as biomarkers, the capacity of ncRNAs to regulate gene expression in general and in gene networks involved in cancer cell transformation in particular, make them a highly attractive therapy against CRC. However, several challenges should be overcome for their use in the clinic such as lack of reliable delivery methods, limited effective vector for ncRNA delivery inside cells, lack of optimal dosage regimes, and determination of side effect. In consequence, novel effective and stable strategies for genome editing, as well as more efficient and less toxic gene therapy delivery systems need to be developed before ncRNA can constitute a potential treatment for CRC.

### 3.3. Gut Microbiome in CRC

The human body contains more than 100 trillion microbes [119], most of which are hosted in the gut, although there are different communities residing in a vast range of body niches [120]. This population is called the microbiome and comprises a wide variety of microorganisms, including archaea, viruses and fungi, although anaerobic bacteria is the most studied group since they are the most abundant [121,122,123]. These microbial communities are acquired at birth [124] and are essential for maintaining body homeostasis.

The gut microbiome comprises an estimated microorganism load of 10^13^–10^14^ belonging to more than 1000 different bacterial species. The interaction between the host and the microbiome is dynamic and controlled by a huge number of genetic and environmental factors, such as age, geography, alcohol or drug intake and diet [125,126,127,128]. Thus, although intestinal microbiota have been shown to be individual and variable over time, only two predominant phyla, namely *Firmicutes* and *Bacteroidetes*, comprise over 90% of all endogenous bacteria present in healthy adults. Other members of the normal colonic microbiome include *Eubacterium*, *Bifidobacterium*, *Fusobacterium*, *Lactobacilli*, *Enterococci*, *Streptococci* or *Enterobacteriaceae* [129,130,131]. The composition of the microbiome is mainly studied using rRNA sequences as traditional culture methods are inadequate in most cases [131,132,133].

Intestinal microorganisms play a critical role in human physiology and metabolism, with their responsibilities including the synthesis of essential vitamins like vitamin K, the extraction of energy from indigestible carbohydrates such as pectins, modulation of the human immune system and the prevention of colonization by enteropathogenic bacteria [119,134,135,136]. In return, these commensal bacteria take advantage of a unique environment with an adequate pH range and oxygen concentration which is also abundant in nutrients.

Given its vast importance, it is not surprising that alterations in normal flora cause serious problems. For instance, the condition known as dysbiosis, in which the natural relationship between the host and the intestinal microbiota is disrupted [137,138,139,140], is considered to be one of the most probable causes of inflammatory bowel disease (IBD) [141,142,143,144] or colorectal cancer (CRC) [142,144] [145,146,147]. Many factors, such as antibiotic treatment or some types of diet, are known to be involved in the development of dysbiosis [139,148,149].

### 3.4. Dysbiosis and Colorectal Cancer: Breaking the Mutualism

Although it is not yet clear how dysbiosis could induce colonic carcinogenesis, chronic inflammation appears to be the main mechanism. This hypothesis is supported by the fact that many types of cancer are caused by chronic inflammation [150,151]. For example, inflammatory bowel diseases (IBD) are linked to an increased risk of colon cancer [144,152]. The first stages of these diseases involve an alteration to the normal flora, which results in activation of the immune system, thus giving rise to the inflammation that characterizes IBD. As IBD patients have a higher probability of suffering CRC, and dysbiosis has been observed in some cases [142,143,144], it is possible to assume that IBD-related CRC is driven by a previous dysbiosis stage.

As a result, the microbiome has started to be considered one of the prime suspects responsible for the onset and/or evolution of colonic carcinogenesis. This research field is based on the differences found in microbial signature between CRC patients and healthy populations. Indeed, next-generation sequencing methods based on 16s rRNA have revealed an enrichment in proinflammatory bacteria, such as *Fusobacterium*, which is also overrepresented in other diseases (for example IBD), as well as a lower abundance of butyrate producers, such as protective bifidobacteria [145,147].

It is thought that proinflammatory bacteria could inhibit, disturb or exacerbate normal host responses, thus leading to abnormal apoptosis, cell proliferation and inflammation. Another proposed mechanism for the onset of CRC is the presence of bacterial secondary metabolites, such as reactive oxygen intermediates or some kinds of toxins, which can damage host DNA and induce cell transformation [153,154,155,156,157]. However, it remains unknown whether a specific bacterium, a microbial community, or both, acting sequentially or synergistically, are responsible for CRC. A deeper understanding of the role of microbiota in the onset of CRC could result in an important prevention tool, since analysis of microbial population in feces would help to predict the risk of suffering from CRC. Moreover, the results of these studies might lead to the establishment of a set of lifestyle changes that contribute to reducing the risk of developing CRC. Therefore, with the coordinated action of oncologists and endocrinologists, the number of patients affected by CRC could be drastically reduced.

Despite this, researchers have focused on some members of the microbiota as the prime suspects in this multiphase process. In this context, some of the most studied microorganisms are discussed below.

#### 3.4.1. *Fusobacterium* spp

*Fusobacterium* spp comprises a group of anaerobic gram-negative non-spore-forming bacteria commonly found in the oral and intestinal human flora. This bacterial genus displays high heterogeneity and some of its members, such as *F. nucleatum*, the presence of which is increased in CRC samples, have been associated with pathological diseases [158].

Flanagan and colleagues [159] conducted a study using three European cancer cohorts to compare *F. nucleatum* levels in normal tissue and in tumour tissue. They found an enrichment of *F. nucleatum* in tumour tissue from all cancer cohorts, with an over-representation in precancerous adenomas. Interestingly, they also observed a significant correlation between survival and *F. nucleatum* levels in tumour tissue, with higher *F. nucleatum* abundances correlating with a worse prognosis. Similar studies were realized in other parts of the world as, for example Kostic [159] in the United States or Li [160] in China, with analogous results being found in each case.

The role of *F. nucleatum* in tumour development and progression may be related to its proliferative and immunosuppressive effects. The abundance of these bacteria in tumour tissue is directly correlated with an increased production of proinflammatory cytokines such as IL-6, IL-12, IL-17 or TNF-α [161], which is consistent with the upregulation of nuclear factor kappa B (NF-κB) [159]. Similarly, *F. nucleatum* contributes to colon carcinogenesis by releasing short peptides and short-chain fatty acids, which recruit myeloid-derived suppressor cells, thereby suppressing CD4+ T-cell activity. Finally, they can avoid tumour cell lysis mediated by NK cells via their Fap2 protein. Fap2 is able to interact with the T cell immunoglobulin and ITIM (TIGIT domain) receptor of NK cells and, as a result, inhibit their cytotoxic potential [162].

#### 3.4.2. *Bacteroides Fragilis*

Two classes of *B. fragilis* that could colonize the human gut have been described. These classes are distinguished by their ability to produce a heat-labile toxin or not. This toxin, called *B. fragilis* toxin, or simply BFT, is a zinc-dependent metalloprotease that is able to induce inflammation due to its ability to stimulate the production of IL-18, a proinflammatory cytokine. In addition, BFT can clear E-cadherin and, as a consequence, disturb epithelial homeostasis, thus leading to colonic epithelial proliferation and possibly resulting in the onset of colorectal cancer [163,164].

In light of the above, a prevalence of toxigenic *B. fragilis* in the normal flora could be related to a higher risk of malignant transformation of the enterocytes. Toprak and colleagues were the first to compare the abundance of this bacteria between CRC patients and those with no colorectal disease, both groups being Caucasian, finding significantly higher levels of toxigenic *B. fragilis* in the former. Similar results were obtained more recently by other researchers. For example, Boleij and colleagues observed that *BFT* gene prevalence was associated with CRC upon comparing mucosal samples from the Johns Hopkins Hospital [164].

#### 3.4.3. Enteropathogenic *Escherichia coli*

*E. coli* is a commensal bacterium widely found throughout the human gut. However, some strains may be linked to the onset of CRC. Thus, phylogroups B2 and D are commonly found to be responsible for some intestinal diseases because of their production of bacteriocins. Indeed, levels of these strains have been found to be significantly increased in CRC samples [165].

The bacteriocin colibactin, the production of which might lead to a higher risk of malignant transformation, is a hybrid nonribosomal peptide-polyketide encoded by the 54 kDa polyketide synthase (*pks*) genotoxicity island. The pro-tumour properties of this bacteriocin are related to its ability to cause DNA double-strand breaks and chromosomal instability [166,167].

### 3.5. Microbiome and Diet: A Possible Link with CRC

As CRC incidence is rapidly increasing in regions where it was previously not a problem, such as Eastern Asia or Eastern Europe and Mediterranean countries [168,169,170], it is thought that dietary changes are mainly responsible for this trend. Although large quantities of fruit and vegetables used to be consumed in these regions, food consumption habits have recently turned towards a more “Western diet” characterized by a high abundance of red meat and fat [171].

Although meat consumption has increased at the same time as colon cancer incidence, this correlation does not necessarily imply causation. However, some mechanisms that could explain this relation have been proposed, including *N*-nitroso compounds (NOCs), heterocyclic amines and polycyclic aromatic hydrocarbons or heme iron [172]. Nevertheless, one of the most interesting effects of high red meat and processed meat consumption is the consistent changes in gut microbiome composition, which leads to an enrichment in harmful bacteria [173].

In this regard, studies by Ou and colleagues [174], who compared samples from rural Africans with African Americans, revealed that microbiome composition is greatly influenced by dietary patterns. Thus, although both groups belonged to the same ethnicity, their microbiomes were significantly different, with *Prevotella*, *Succinivibrio* and *Oscillospira* predominating in African samples and Bacteroidetes such as *B. fragilis* predominating in African Americans. These differences could be explained by the fact that African Americans tend to eat more red meat and fat than rural Africans. Feng and colleagues [175] took this supposition to the next level: by comparing samples from CRC patients with different dietary patterns. Using a metagenomic analysis, they discovered that those bacteria whose populations were higher in CRC samples (for example *B. massiliensis*, *P. merdae*, *A. finegoldii* or *B. wadsworthia*) were found at lower levels in those subjects whose fruit and vegetable intake was abundant; in contrast, they were elevated in high red meat consumers.

In addition to high meat consumption, a diet rich in fat could also be responsible for increasing CRC risk. Thus, O’Keefe and colleagues [176] studied CRC samples from African Americans and rural Africans and found lower levels of butyrate producers in African Americans compared with rural Africans. As the antitumour properties of butyrate are well established [177,178], this fact could explain the higher incidence in response to a diet rich in fat. Moreover, a high fat intake is also related to higher expression of the microbial genes involved in deconjugation of bile acids and production of secondary bile acids [176]. Secondary bile acids such as lithocholic acid are considered to be tumour-promoting agents due to their ability to induce oxidative stress, DNA damage or mutation [66]. Taken together, these two factors could explain why a high fat intake leads to the onset of colon cancer.

However, if diet is the problem it could also be the solution. Thus, the study of O’Keefe [176], for example, shows that a diet rich in fiber leads to higher butyrate production. Donohoe and colleagues [179] developed a BALB/c inbred mouse model polyassociated with four commensal bacteria plus or minus the butyrate producer *Butyrivibrio fibrisolvens* to show that fiber intake could protect against CRC via microbiome butyrate production. These mice were fed with identical and calorically matched low- or high-fiber diets; as expected, the animals given the higher amount of fiber produced the most butyrate. Upon injection of tumour-promoting agents, mice colonized with *B. fibriosolvens* and fed with the high-fiber diet were less sensitive to tumour development, thus proving the protective effect of butyrate in CRC.

## 4. Recent Advances in CRC Diagnosis and Staging

### 4.1. Use of Biomarkers in CRC

A biomarker is a biological entity that can be used to measure the presence or progression of a particular disease or the effects of treatment. Biomarkers must possess several important characteristics, such as high sensitivity, specificity, and safety, in addition to being easy to measure and useful for establishing an accurate diagnosis and facilitating treatment selection [180].

As noted previously, three major alterations, namely microsatellite instability (MSI), chromosomal instability (CIN) and the CpG island methylator phenotype (CIMP), are found in CRC. These alterations produce modifications in DNA, RNA, proteins or metabolites, which can be measured in the tumour specimen, blood or stool and can therefore be used as biomarkers [181]. Some of the most important biomarkers for CRC, and their clinical use, are summarized in Table 3. In contrast to the methods presently used (colonoscopy, sigmoidoscopy, double contrast barium enema, computer tomographic colonoscopy and fecal blood test (FOBT)), molecular tests are expected to be more specific, sensitive and better tolerated by patients, although further studies are needed for their validation.

The most widely used biomarkers in CRC are currently the determination of MSI and *KRAS* mutations in tumour samples in order to classify the tumour, make a prognosis of the disease and manage therapy [181]. Although other biomarkers, including the determination of FOBT and CEA, are used for diagnosis, they tend to exhibit high specificity but very low sensitivity [182]. This is the main reason why researchers are searching for better molecules for the early diagnosis of CRC. In this context, the most remarkable findings include the use of kits to evaluate the CpG island methylator phenotype, miRNA and gene microarrays which can be detected in stool or blood. Most of these kits are under clinical evaluation and have a promising future.

Considering the limitation of the actual screening methods for CRC—invasiveness, low specificity and sensitivity and high cost—the identification of new molecular biomarkers with predictive and/or prognostic significance in CRC has become an essential issue to improve anti-cancer treatments and patient outcome. Over the past two decades, several molecular biomarkers have been studied and the obtained results are encouraging; however, several drawbacks affect the reliability of the conclusions [145]. First, most of the published studies were retrospective analyses of a single marker or included a small sample size, and, in consequence, predictions lack resolution and reproducibility. Second, data analysis and interpretation remain challenging; data obtained usually lack definition and adequate validation, and are thus not reliable enough to be used in clinical practice. Furthermore, the lack of methodology for standardization, as well as the absence of standardized endogenous controls, make it difficult to quantify and validate the obtained results.

In consequence, although the number of potential biomarkers is large, only the *KRAS* gene has entered routine clinical practice, and it is used as a predictive marker of response to EGFR-targeted therapies in advanced CRC stages. Nevertheless, a great deal of effort is being focused on this issue, and despite all their actual disadvantages, the use of biomarkers have a promising future in the diagnosis and prognosis of CRC, as well as in the development of personalized and targeted therapy.

### 4.2. Gene-Expression Profiling (GEP)

Gene-expression profiling (GEP)-based studies compare gene expression in normal and tumoural tissue samples or samples from different stages of the disease [210]. It is thought that these comparisons can provide useful information about the prognosis of the disease and the most suitable treatment for each patient. Several GEP techniques are currently available from CLIA-certified laboratories (Table 4), with these differing in terms of the type of assay used and the number of genes studied.

However, despite the number of GEP assays that have appeared in the last years, and their potential benefits for both patients (reduction of harm and side effects of adjuvant therapies) and society (lower care costs for patients who will not benefit from adjuvant therapies), the National Comprehensive Cancer Network (NCCN, 2015) has stated that there is still insufficient evidence regarding their predictive value in terms of the potential benefit of chemotherapy to any of the available multigene assays. Moreover, the NCCN has determined that not enough data are available to base the choice of adjuvant therapy on these assays, hence further studies in this field should be conducted to address their diagnostic and clinical validity.

## 5. Novel Therapies for the Treatment of CRC

### 5.1. Current Treatments for CRC

The choice of first-line treatment for CRC patients currently involves a multimodal approach based on tumour-related characteristics (e.g., number and localization of metastases, tumour progression, presence or absence of biochemical markers, etc.) and patient-related factors (e.g., co-morbidity, prognosis, etc.). In practice, all these aspects are used to classify CRC patients into one of four different risk groups that will be used to guide the treatment strategy: Group 0: Patients with no metastatic disease or with resectable liver or lung metastases and lack of poor prognostic signs (e.g., relapse during adjuvant treatment). In this case, the recommended treatment consists of surgical resection of the metastasis. Chemotherapy has not been found to provide a great advantage in the overall survival of this group; Group 1: Patients with potentially resectable metastatic disease. These patients are initially treated with induction chemotherapy to reduce the number and size of the metastases and enable subsequent surgical resection. Recommended chemotherapy for these cases comprises cytotoxic doublet or triplet, which may be combined with anti-VEGF or anti-EGFR strategies in KRAS wild-type tumours [222,223]; Group 2: Patients with disseminated unresectable disease. Treatment selected for this group of patients will be palliative rather than curative, with the main intention of reducing the symptoms, aggressiveness and extension of the disease. As such, the first-line treatment selected should induce metastatic regression in a short time. To that end, the preferred option usually comprises a cytotoxic doublet in combination with a targeted agent (anti-VEGF or anti-EGFR strategies). In oligometastatic patients who respond to treatment, additional ablative methods may be considered to increase the progression-free interval. If ablative methods cannot be used, de-escalation of the initial combination should be studied as a maintenance treatment. In certain cases, complete discontinuation of the treatment can be considered [222,223]; Group 3: Patients with unresectable disease and lack of intensive or sequential treatment: In patients lacking symptoms with low risk of deterioration, the purpose of the treatment will be to prevent tumour progression and increase treatment-free life. The most commonly used strategies comprise a fluoropyrimidine as cytotoxic agent combined, or not, with a biological targeted agent [222,223].

As mentioned above, most CRC patients with metastatic disease are treated with a combination of cytotoxic and targeted biological agents. First-line chemotherapy with palliative purposes comprises fluoropyrimidines (e.g., 5-fluorouracil (5-FU) or capecitabine) alone or combined with leucovorin (LV) as well as other cytotoxic agents, such as oxaliplatin (5-FU/LV/oxaliplatin (FOLFOX) and capecitabine/LV/oxaliplatin (CAPOX)) or irinotecan (5-FU/LV/irinotecan (FOLFIRI)). The use of leucovorin reduces the toxicity of the treatment, whereas the addition of other cytotoxic agents has been shown to increase the response rate and progression-free survival, although the toxic effects of the treatment are also intensified [223,224].

Second-line chemotherapy will be offered to patients with good organ function and should be selected according to a refractory-based regimen. Second-line treatment for patients refractory to irinotecan will consist of an oxaliplatin-containing combination such as FOLFOX or CAPOX, whereas patients refractory to FOLFOX or CAPOX will be treated with irinotecan monotherapy or FOLFIRI [224].

The optimal duration of chemotherapy treatment depends on each case, with three different options being available: fixed treatment for 3–6 months, induction treatment followed by a maintenance treatment, or treatment until toxicity or progression [223].

In addition to chemotherapy, monoclonal antibodies or proteins against vascular endothelial growth factor (VEGF) and epidermal growth receptor (EGFR) combined with traditional chemotherapy have been demonstrated to improve the outcome of mCRC [223]. The most commonly used anti-VEGF strategies are the monoclonal antibody Bevacizumab, which targets circulating VEGF-A, and the recombinant fusion protein Aflibercept, which blocks VEGF-A, VEGF-B and placental growth factors. When combined with cytotoxic agents, they represent a first-line treatment for almost all CRC patients. In contrast, anti-EGFR treatment can only be used in the absence of KRAS mutations, either as a single agent or combined with cytotoxic molecules. The most important anti-EGFR agents comprise monoclonal antibodies such as Cetoximab or Panitumumab [223].

Besides traditional chemotherapy, alternative therapies are being studied with the aim of increasing treatment efficacy and reducing side effects as well as the risk of developing secondary tumours. The most important research lines currently in progress are the use of agarose tumour macrobeads, anti-inflammatory drugs, probiotics, and gold-based drugs.

### 5.2. Agarose Macrobeads

Organs and tumours grow following a Gompertzian curve, a decremented exponential curve that approaches an asymptote or decreases as enlarged. This evidence suggests that tumours, like organs, are susceptible to both positive and negative growth-regulatory controls [225]. The positive regulation of tumour growth is well established and there is a large body of evidence in the literature to support this. For instance, it has been shown that the partial surgical removal of a tumour often induces tumour progression caused by a phenomenon called “compensatory hyperplasia” [226]. Similarly, biological signals indicating the presence of a tumour mass reduce or stop tumour growth even when that mass of cells is not present [226]. This hypothesis constitutes the basis for the use of hydrophilic agarose macrobead culture cells for CRC outgrowth.

Agarose macrobeads consist of two concentric layers of agarose, which create an inner space where cancer cells can be contained [227,228]. Various tumour cell lines have the ability to form colonies after encapsulation in agarose macrobeads, but for this application in particular, RENCA cells (a mouse renal cortical adenocarcinoma cell line) have been selected. After encapsulation, these cells form one-cell colonies that expand in size up to colonies containing several hundreds of cells. As the colonies enlarge, their growth slows until they acquire a stable size, which occurs around 6 to 24 months after encapsulation. During this time, the encapsulated cells undergo a transformation process in which at least two subpopulations of cells are selected to form the tumour colonies [227]. The growth-restrictive agarose environment induces the production of tumour inhibitory molecules, which are able to inhibit the proliferation of non-encapsulated cancer cells both in vitro and in vivo [227]. These include the proteins Gelsoin (GSN), Fibulin (FBLN1), nucleolin (NCL), Prosaposin (PSAP), pigment epithelium-derived factor (PEDF), Serpine1 (Serbp1), secreted protein acidic and rich in cysteine (SPARC), tissue inhibitor of metalloproteinase 2 (TIMP2), phosphatidyl ethanolamine-binding protein (PEBP1), and peroxiredoxin 1 (PRDX1) [227]. Although the mechanism of growth inhibition by RENCA macrobeads is not yet clear, it is thought that all these secreted proteins generate multiple signals, which end up increasing the cell-cycle time of the exposed cancer cells. In particular, the results of different experiments demonstrate that RENCA macrobeads extend the S-phase cycle time and decrease the mitosis number. As the mechanism of action of RENCA macrobeads is quite nonspecific, this regulatory system is applicable to many epithelial-derived tumour types from different species and cell lines [227].

RENCA macrobeads have been tested in advanced epithelial-derived cancer in a phase I/II clinical trial. In general, RENCA macrobeads were well tolerated by patients. Some common adverse effects were fatigue and anorexia lasting from a few days to three weeks. Other less-common adverse effects were abdominal pain, constipation, pyrexia, nausea, vomiting, dyspnea, localized fluid accumulation around the MBs, ascites, abdominal distension and peripheral oedema. Most of the treated patients showed a positive response to treatment, including a reduction in tumour markers, disease stabilization, reduction of pain and improvement in quality of life [51,229]. A phase II/III clinical trial is currently underway and the future perspectives for this treatment are quite encouraging.

### 5.3. Anti-Inflammatory Drugs

Chronic inflammation, a common feature in colorectal cancer, is caused by immune cells and their products (cytokines and chemokines), reactive oxygen and nitrogen species (ROS and RNS) and also some arachidonic acid derivatives, mainly produced via the cyclooxygenase (COX) and lipoxygenase (LOX) pathways. This inflammation state collaborates in tumour growth, proliferation, invasion and resistance [230].

Since inflammation is known to play an important part in the generation and progression of colorectal cancer, many anti-inflammatory drugs have become important in the prevention and treatment of CRC. Most of the anti-inflammatory agents used are non-steroidal anti-inflammatory drugs (NSAIDs), which inhibit COX enzymes, thereby blocking the synthesis of arachidonic acid derivatives. For example, aspirin has demonstrated good results in the prevention of CRC, reducing risk by up to 50% [231]. Another example of an NSAID used in CRC-derived inflammation is sulindac [232], which has been shown to reduce colorectal cancer inflammation. Moreover, sulindac combined with atorvastatin has even been proved to inhibit tumour growth [233].

Despite their anti-inflammatory properties, NSAIDs have negative side effects such as gastrointestinal ulcerations or kidney damage [234], hence they are mostly used to prevent CRC in high-risk patients, such as those with Crohn’s disease, ulcerative colitis or FAP, rather than for treatment. To avoid these side effects, a new kind of NSAIDs that specifically inhibits cyclooxygenase 2 (COXibs) has been developed to both prevent and treat CRC [235]. Although they do not affect the gastrointestinal system, some of these compounds, such as rofecoxib, which was withdrawn from the market in 2004, can produce cardiovascular toxicity [236]. The most important drug within this group of COXibs is celecoxib, which has been demonstrated to prevent colorectal cancer and reduce adenomas, even those in advanced states. Moreover, celecoxib does not present adverse gastrointestinal effects [237] and does not produce cardiovascular problems [238]. In addition, new formulations are being studied to further reduce the known side effects. One example of these new drugs is celecoxib microbeads, which only target colon cells. This formulation is currently being tested using in vitro models, with studies in animal models expected in the near future [239]. Celecoxib has also been tested in combination with curcumin, a product of curcuma, obtaining a synergistic anticancer effect [240].

### 5.4. Probiotics

Having demonstrated the role of microbiota in colorectal cancer onset and progression, it is legitimate to suppose that it could be switched to a “non-carcinogenic” microbiome, thereby avoiding the tumourigenic process. In that regard, probiotics are a potential treatment for CRC, being important for its prevention, and can be used as adjuvants for conventional treatments.

Probiotics are viable microorganisms which, when administered in appropriate quantities, confer a health benefit on the host. Lactic acid bacteria, including *Lactobacillus*, *Streptococcus*, *Enterococcus*, *Lactococcus*, *Bifidobacterium* and *Leuconostoc*, are the most studied probiotics in CRC treatment [241,242,243].

The protective role of probiotics is based on the hypothesis that dysbiosis is the main cause of CRC. As has been discussed previously, differences in the microbial signature of CRC patients and healthy controls lead us to the hypothesis that probiotics could help to restore the normal flora and hence avoid CRC. In order to assess this hypothesis, Pala and colleagues [244] designed a study to correlate yogurt consumption and CRC risk involving 45,241 healthy volunteers whose dietary patterns and other lifestyle aspects were carefully analysed. These authors found that yogurt intake was directly related to a lower CRC risk when considering other lifestyle variables as well, such as whether yogurt was ingested alone or in combination with other dairy products.

Insights into the mechanism of action of probiotics have mainly been obtained using animal models given the difficulty of working with human subjects. For example, Chen and colleagues [245] used a mouse model of colon cancer to investigate the role of orally administered *Lactobacillus acidophilus* and found that these bacteria could reduce the harmfulness of the disease by inducing apoptosis. According to results obtained by Choi and colleagues, this ability to promote cell death might be related to soluble polysaccharides [246].

Apart from their apoptosis-enhancing activity, probiotics play an important role in CRC prevention due to their antioxidant potential. Thus, Sah and colleagues [247] demonstrated that some probiotic strains present in yogurts are able to produce antioxidant peptides with free radical scavenging activity, meaning that they may reduce oxidative stress in the lumen and, as a consequence, prevent or delay the onset of CRC.

### 5.5. Functional Foods

Reactive oxygen species (ROS) are oxygen molecules with a missing or unpaired electron produced as a consequence of cellular metabolism. At low concentrations, ROS play a physiological role in the defence against pathogens, the mitogenic response and in different molecular pathways [248,249,250]. However, an excess of these reactive compounds, which can be induced by environmental pollution and tobacco or drug consumption, amongst other stressors, can damage different cell structures via the oxidation of lipids, proteins and DNA [251]. These situations have been associated with several human diseases, such as arthritis or cancer [252]. As a consequence, maintaining and re-establishing the redox balance is critical for the correct functioning of the whole organism, hence redox and antioxidant systems are amongst the most promising targets in functional food science [253,254,255]. In this context, polyphenols obtained from natural sources have emerged as a new strategy to protect cells against oxidative stress.

Polyphenolic compounds are the most abundant secondary metabolites found in plants [256]. Many foods and food material contain polyphenols, including cereals and legumes (barley, corn, nuts, oats, rice, sorghum, wheat, beans, and pulses), oilseeds (rapeseed, canola, flaxseed, and olive seeds), fruit and vegetables, and beverages (fruit juices, tea, coffee, cocoa, beer, and wine) [256,257]. All of them possess an aromatic ring bearing one or more hydroxyl groups [255]. These polyphenolic substances or polyphenols include many classes of compounds, ranging from phenolic acids to coloured anthocyanins, simple flavonoids and complex flavonoids [258]. In addition to anticarcinogenic effects, all these polyphenols have a strong antioxidant power, thereby decreasing the risk of cancer [259]. Indeed, many polyphenols have been shown to have chemoprotective, antiproliferative, antioxidative and estrogenic/antiestrogenic activity in addition to inducing cell-cycle arrest or apoptosis and the detoxification of enzymes. They are also known to regulate the host’s immune system and changes in cellular signalling [260]. In particular, proanthocyanidins, flavonoids, resveratrol, tannins, epigallocatechin-3-gallate, gallic acid, anthocyanins and some plant extracts have shown protective effects in some cancer models [261,262].

### 5.6. Metal-Based Drugs for CRC Treatment

The use of metals for therapeutic purposes dates back to ancient times. For example, cinnabar powder, a derivative of mercury, was widely used in traditional Chinese and Indian medicine [263]; silver sulfadiazine is commonly found in topical creams for burn treatment [264], and Tianmai Xiaoke Tablet, the main ingredient of which is chromium picolinate, is used in China to treat type 2 diabetes [265]. However, one of the most important achievements for inorganic chemistry in medicine is the discovery of cisplatin. The anticancer properties of *cis*-PtCl_2_(NH_3_)_2_, or cisplatin [266], were discovered accidentally in the 1960s. Since then, many other metal-containing compounds have been developed in order to treat cancer [267,268,269,270]. Herein we will discuss the most promising candidates, namely platinum and gold, and their use in colorectal cancer chemotherapy.

#### 5.6.1. Platinum

Cisplatin was the pioneer in the use of metals in chemotherapy. This compound has been found to be effective against various cancer lines, including testicular, ovarian and solid tumours of the head and neck [266,267,268,269,270,271]. Its success arises due to its mechanism of action: cisplatin is able to bind DNA and, as a result, to induce apoptosis. Once inside the cell, cisplatin reacts with DNA at the N7 position of the major groove guanines, which is the most exposed and nucleophilic site. Mono- and bifunctional adducts are then formed between cisplatin and DNA as a result of inter- and intra-crosslinks [272,273].

Despite its activity, the use of cisplatin results in serious side effects due to its mechanism of action. The most relevant side effects are kidney damage and hearing loss. Nephrotoxicity is related to the accumulation of cisplatin in the kidney proximal tubule [274,275], whereas ototoxicity arises due to the death of cochlear sensory hair cells resulting from the increase in reactive oxygen species [274,275,276]. In addition, an increase in tumour resistance to cisplatin treatment has been observed. The most widespread resistance mechanism involves an increase in the repair of cisplatin-DNA adducts. An increase in cytosolic drug inactivation and a reduction in drug uptake are other possibilities [277,278].

In order to maintain the efficacy of cisplatin without its shortcomings, numerous analogues, amongst which oxaliplatin and carboplatin stand out, have been developed. Thus, oxaliplatin is one of the most common drugs used in colorectal cancer chemotherapy. The importance of oxaliplatin, or *trans*-l-diaminocyclohexaneoxalatoplatinum, resides in the absence of cross-resistance with cisplatin, thus allowing it to be used when a tumour shows resistance to this drug. This absence of cross-resistance with cisplatin is due to the different mismatch-repair proteins that recognize each kind of adduct, and also explains their different side effects [279]. However, what is more remarkable is that oxaliplatin does not induce ototoxicity and nephrotoxicity. Oxaliplatin is commonly administered in combination with infusional 5-fluorouracil/leucovorin, a regime called FOLFOX [280]. FOLFOX has greatly improved the response rate: 5-fluorouracil treatment gives a response rate of 20%, whereas this increases to 50% when combined with oxaliplatin [281]. The 5-fluorouracil and oxaliplatin combination also provides a better survival rate in metastatic patients [281,282].

#### 5.6.2. Gold

Many metal-based anticancer drugs containing gold(I) or gold(III) have been designed over the past few decades. However, the structures of these compounds are quite different as the gold atom can be coordinated to phosphines, carbine ligands, porphyrinates or dithiocarbamates in order to improve its antitumour effect and other properties [267,283,284,285,286]. One of the best characterized gold-containing anticancer drug is known as *auranofin*. [2,3,4,6-Tetra-*o*-acetyl-1-thio-β-d-glycopyranosato(triethylphosphine)gold], is a gold(I) compound containing phosphine and thiol ligands traditionally used as an antirheumatic drug [287]. The mechanism of action of *auranofin* involves the inhibition of thioredoxin reductase, which leads to an increase in reactive oxygen species that causes oxidative stress and finally triggers intrinsic apoptosis [288]. As a result, *auranofin* is able to induce apoptosis even in cisplatin-resistant cancer cells [289].

As the gold atom included in *auranofin* is responsible for thioredoxin reductase inhibition, other gold-containing drugs are also able to induce apoptosis in cancer cell [290,291,292]. Gold atoms are strongly attracted to thiol and selenol groups and can therefore bind selenium-dependent proteins such as thioredoxin reductase, which is upregulated in some kinds of cancers, such as colorectal cancer, and is directly implicated in tumour progression and survival [293], meaning that inhibition thereof triggers cancer cell death [294].

Inhibition of thioredoxin reductase is not the only mechanism that gold-containing drugs can use against colorectal cancer. Thus, *auranofin* can inhibit the ubiquitin-proteasome system [288,295], which is critical for colon cancer cell homeostasis [296]. Once again, this affinity is extensible to all gold-containing drugs. In particular, gold(I) derivatives disrupt the redox balance, increasing ROS levels inside the cell and altering the mitochondrial membrane potential, which induces activation of the apoptosis cascade and results in controlled cell death, rather than affecting nucleic acids [297] [298,299,300,301,302,303].

## 6. Discussion and Future Perspectives

Because of its high incidence and mortality rate worldwide, colorectal cancer (CRC) has become a global public health problem. Herein we have reviewed the latest discoveries in the study of CRC research, as well as the newest findings in diagnostic and treatment methodologies in order to provide researchers and clinicians with an updated vision of the key insights into this disease.

The onset and development of CRC is induced by a combination of genetic and environmental factors, whose study is essential for the establishment of new prevention strategies—one of the most important lines of action to stop the increase in its incidence. Over the last decade, the major colon cancer genes have been identified and their pathogenic variants have been associated with a high susceptibility to CRC. Moreover, the study of their inheritance patterns led to the discovery of Hereditary Colorectal Cancer Syndromes, which can now be diagnosed, as well as to special risk management programs and genetic counseling that can be applied to patients and their relatives, reducing their risk of suffering from this disease. However, familial clustering of colon cancer also occurs outside of the setting of well-characterized colon cancer family syndromes; therefore, different epidemiological studies are being carried out with the aim to identify polymorphisms underlying susceptibility to CRC in different populations. Results obtained in those studies can set the foundation for a new generation of genetic screening tests.

Although most of the major cancer genes involved in CRC have been well characterized, the influence of additional environmental factors in this disease remains undefined. In this context, a more in-depth study of the relationship between diet, microbiota and CRC is needed. The results of these studies might lead to the establishment of a set of lifestyle changes that contribute to reducing the risk of developing CRC. Moreover, since the composition of microbiota also seems to influence the development of CRC, adjuvant therapies based on probiotics and prebiotics are being studied to improve the response to traditional chemotherapeutic agents and to reduce dosage and frequency of drug administration, resulting in an improvement of the patient’s quality of life.

Similarly, the development and implementation of new specific and more sensitive biomarkers in the foreseeable future will improve diagnostic strategies, thus allowing clinicians to detect CRC cases in the earliest stages of the disease and hence to improve the prognosis of thousands of patients. Currently, only the determination of MSI and KRAS mutations in tumour samples are in use with diagnostic and therapy management purposes. For early diagnosis of CRC, different tests based on miRNA expression, gene microarrays, and CpG island methylation phenotype are under evaluation and, although they have a promising future, further studies of larger populations are needed for their validation.

In the context of treatment, personalized medicine is fast becoming an indispensable tool. Thus, it is necessary to perform an in-depth analysis of the tumour features of each patient to find the most appropriate treatment.

Finally, an extensive part of current research into CRC is focused on the development of new therapies that are less aggressive and more effective than conventional ones. Discoveries in this area and their clinical implementation will improve the overall survival and quality of life of CRC patients in the future.

## Figures and Tables

**Table 1:** miRNAs involved in colorectal cancer (CRC).

miRNA Name	Target	Function of miRNA	References
miR-34a	SIRT1, FMNL2 and E2F5	Inhibition and induction of p53 acetylation	[53,54]
miR143	DNMT, KRAS	Induction of cell proliferation	[55]
miR135	APC	Suppression of WNT pathway	[55]
miR-29	DNMT 3A and 3B	Reduction of methylation	[56]
miR-21	PDCD4	Invasion and metastasis promotion	[57]
miR-345	BAG	Induction of cell proliferation and invasion	[58]
miR-148b	CCK2R	Induction of cell proliferation	[59]
Let-7c	KRAS, MMP11 and PBX3	Metastasis induction	[60]
Let-7a	Np95 ICBP90 RING finger	Induction of cell proliferation	[61]
miR-499-5p	FOXO4 and PDCD4	Induction of metastasis	[62]
miR-92	KLF4	Promotion of cell growth and migration	[63]
miR-126	SPRED1, PIK2R2/P85-β	Inhibition of cell proliferation, migration and invasion	[64]
miR-320	FOXO4 and PDCD4	Inhibition of cell proliferation	[65]
miR-200 family	JNK2	Inhibition of tumour growth and metastasis and induction of sensitivity to chemotherapeutic drugs	[66]
miR-9	TM4SF1	Suppression of cell migration and invasion	[67]
miR-503	calcium-sensing receptor	Induction of proliferation migration and invasion	[68]
miR-222	MST3	Induction of invasion and migration	[69]
miR-181b	RASSF1A	Induce proliferation and enhance cell survival	[70]
miR-497	VEGFA	Inhibition of invasion and metastasis	[71]
miR-152	PIK3R3	Tumour suppressor	[72]
miR-187	SOX4, NT5E and PTK6	Inactivation of TGF-β pathway and prevention of EMT (epithelial to mesenchymal transition)	[73]
miR-519	Orai1	Tumour suppression	[74]
miR-155	HMG-box transcription factor 1	Tumour suppressor by induction of WNT/β-catenin pathway	[75]
miR-497	KSR1	Tumour growth inhibition and enhancement of chemo sensitivity	[76]
miR-375	Bcl-2	Inhibition of tumour progression	[77]
miR-1246	CCNG2	Induction of cell growth and metastasis	[78]
miR-140-5p	VEGFA	Inhibition of tumour progression	[78]
miR-144	GSPT1	Inhibition of proliferation and migration	[79]
miR-638	Phospholipase D1	Inhibition of cell proliferation	[80]
miR-99b-5p	mTOR	Inhibition of metastasis formation	[66]
miR-101	SphK1	Inhibition of cell growth and increase of paclitaxel chemo-sensitivity	[81]
miR-20a	TIMP-2	Induction of epithelial-to-mesenchymal transition (EMT)	[82]
miR-409-3p	GAB1	Inhibition of tumour progression and metastasis	[83]

**Table 2:** LncRNAs involved in CRC.

LncRNA	Locus	Size (kB)	Dysfunction Type	Normal Function	Contribution to Cancer	References
H19	Chr11p15.5	2.3	Overexpression	Regulation of growth during development targeting Igf2	Downregulation of the tumour suppressor RB	[88,89,90]
HOTAIR	Chr12q13.3	2.2	Overexpression	Epigenetic silencing of gene expression	Reprogramming of chromatin state and induction of metastatic progression	[91,92]
MALAT1	Chr11q13.1	7	Overexpression	Alternative splicing regulation	Increase of abnormal mitosis, invasion and metastasis and induction of cell death resistance	[93]
HULC	Chr6p24.3	0.5	Overexpression	Sponge for miR-372 and indirect upregulation of PKA and activation of CREB	Upregulation of Prkacb (catalytic subunit of PKA)	[94]
MEG3	Chr14q32	1.6–1.8	Downregulation	Tumour suppressor. It activates p53, inhibits cell proliferation and controls gene imprinting	Downregulation of p53, apoptosis inhibition and induction of proliferation	[95]
CCAT1	Chr8q24.21	2.6	Overexpression	Enhancer region for cMYC. Maintenance of the chromatin looping between MYC promoter and its enhancer	Induction of MYC expression and inhibition of G1 arrest. Enhancement cell proliferation and migration	[96,97]
CCAT2	Chr8q24	0.4	Overexpression SNP rs6983267	Upregulation of MYC and enhancement of WNT signalling pathway through TCF7L2	Induction of cell proliferation, invasion and chromosomal instability	[98]
CRNDE	Chr16:hCG_1815491	10	Overexpression	Scaffold for regulatory complexes	Contribution to Warburg effect. Increase of CRC risk	[99]
LOC285194	Chr3q13.31	2.1	Downregulation	Unknown	Decrease of cell migration and metastasis	[100]
OCC-1	Chr12121.1	1.2–1.3	Overexpression	Unknown	Induction of cell proliferation and apoptosis resistance	[101]
lincRNA-p21	-	3.1	Downregulation	Binds to hnRNP-K and repress genes transcriptionally regulated by p53. It is necessary for p53-dependent apoptotic induction but not for cell-cycle arrest	Induction of apoptosis evasion, invasion and enhancement of Warburg effect	[102]
LIT1	Chr11q15.5	91	Loss of imprinting	Organization of a tissue/lineage-specific nuclear domain involved in epigenetic silencing of the Kcnq1 imprinting control region	Unknown	[103]
PTENP1	Chr9q13.3	3.9	Downregulation	Decoy for miRNA targeting PTEN	Reduction of PTEN level and enhancement of cell growth	[104]
MYLKP1	Chr3p12.3	106	Overexpression	Pseudogen	Induction of proliferation	[105]
pou5f1p1 (OCT4)	Chr8q24	0.4	Overexpression	Pseudogen	Increased of risk of CRC	[106]
UCA1	Chr19p13.12	1.4, 2.2, 2.7	Overexpression	Embryonic development	Induction of resistance to drug-induced apoptosis	[107]
PCAT1	Chr8p24	1.9	Overexpression	BRCA2 inhibition	Regulation of cell response to genotoxic stress and impairing of DNA damage repair. High levels are associated with poorer survival rate	[108,109,110]
PRNCR1	Chr8p24	13	Overexpression	Binding to the androgen receptor and enhancement of both androgen-receptor-mediated gene activation and proliferation	Increase of cell proliferation	[72,111,112]
LET	Chr15q24.1	2.3	Downregulation	Downregulation of hypoxia signalling by decreasing HIF1 stability. Induction of NF90 ubiquitination and Go/G1 arrest	Induction of metastasis	[113]
ncRAN	Chr17q25.1	2.3	Overexpression	Unknown	Enhancement of cell migration and invasion	[114]
PVT1	Chr8p24.21	>300	Overexpression	Regulation of C-MYC	Anti-apoptotic activity. Increase of cell proliferation and cell-cycle progression	[115,116]

**Table 3:** Current biomarkers for CRC.

Molecular Marker Type	Biomarker	Contribution to Cancer	Predictive Use	Samples Used for the Test	Status	References
DNA	Microsatellite instability (MSI) test. Panel of mononucleotide marker (Bat-25, Bat-26, NR-21, NR-24, MONO-27), ≥30% of unstable loci are considered MSI tumours.	Accumulation of alteration in highly repeated DNA sequences	For MSI+ tumours: Prognosis: good, aggressively: low, treatment: lack of response to 5-FU, good response to irinotecan	Tumour-based samples	In use	[183,184,185]
-	KRAS, NRAS	Proliferation enhancement through EGFR-signalling activation	If mutated: Prognosis: bad and poor survival (codon 12 and 13). Treatment: limited response to EGFR	Tumour-based samples, stool	In use for tumour-based samples and under evaluation for stool	[186,187,188]
	BRAF	Proliferation enhancement through EGFR-signalling activation	If mutated: Classification of CRC: sporadic, Prognosis: poor, Treatment: limited response to EGFR-targeted therapy.	Tumour-based samples	In use	[189,190,191]
	CpG Island Methylator Phenotype. e.g., Vimentin methylation.	Transcriptional regulation which lead to colorectal carcinogenesis	Classification of CRC in CIMP, Presence of BRAF mutations	Tumour-based samples, stool, blood samples	Under evaluation in tumour samples and in use for stool	[192,193]
	Integrity of cell-free DNA (cfDNA)	Apoptosis	Diagnosis and monitoring	Blood sample	Under evaluation	[194]
RNA	gene microarray and gene panels of RNA	Unknown	CRC diagnosis evaluation of relapse risk	Tumour-based samples, stool, blood	Clinical validation	[195,196]
	miRNA biomarker panel. e.g., miR-21, miR-106a	Unknown	Diagnosis and prognosis	Tumour-based samples, stool, blood	Clinical validation	[197,198]
	EGFR ligand biomarker panel (amphiregulin, epiregulin, DUSP6 and SLC26A3)	Proliferation enhancement through EGFR-signalling activation	Response to EGFR-targeted therapy	Tumour-based samples	Under evaluation	[199,200,201]
Protein	Tumour-specific protein determination. e.g., Calprotectin, CEA, DAF, CA19-9	Unknown	Diagnosis, prognosis, monitoring	Stool, blood	Clinical validation	[202,203,204,205,206]
Others	Circulating nucleic acids, proteins and tumour cells	Unknown	Diagnosis, monitoring	Blood	Clinical validation	[207,208,209]

**Table 4:** Current Gene-Expression Profiling (GEP) for CRC.

Assay	Name of the Assay	DNA Markers Used	Type of Test	References
ColonSentry^®^ (GeneNews, (Toronto, ON, Canada))	Determination of relative risk to suffer CRC	ANXA3, CLECD4, LMNB1, PRRG4, TNFAIP6, VNN1, IL2RB	qRT-PCR	[211,212]
Oncotype DX^®^ Colon Cancer Assay (Genomic Health, Inc., Redwood City, CA, USA)	Prediction of recurrence in individuals with stage II CRC following surgery	7 Genes associated with CRC recurrence (Ki-67, C-MYC, MYBL2, FAP, BGN, INHBA, GADD45B,) and 5 reference genes (ATPSE, PGK1, GPX1, UBB, VDAC2)	qRT-PCR	[213,214]
ColoPrint^®^ (Agendia, BV, Amsterdam, The Netherland)	Determination of risk of distant recurrence of the disease in individuals with stage II and III colon cancer	MCTP1, LAMA3, CTSC, PYROXD1, EDEM1, IL2RB, ZNF697, SLC6A11, IL2RA, CYFIP2, PIM3, LIF, PLIN3, HSD3B1, ZBED4, PPARA, THNSL2, CA4388O2	Microarray	[215,216]
Colorectal Cancer DSA^®^ (Almac Diagnostics, Craigavon, UK)	Risk of CRC recurrence within 5 years	ABCC3, FGF1, ISG15, OXNAD1, PPP2CA, PRKACB, TP53INP1, ARHGAP18, BEST1, FKBP5, KITLG, LAMP3, MRPS31, NPM3	Microarray	[217]
GeneFx Colon^®^ (Precision Therapeutics, Pittsburgh, PA, USA)	Risk of CRC recurrence within 5 years	-	634-transcript DNA microarray-based gene signature	[218]
OncoDefender-CRC^®^ (Everist Genomics, Ann Arbor, MI, USA)	Risk of recurrence of cancer in individuals of stage I or II colon cancer or stage I rectal cancer.	BMI1, ETV6, H3F3B, RPS10	qRT-PCR	[219,220]
Previstage (DiagnoCure, Quebec City, QC, Canada)	Identification of patients with low risk of recurrence	Quantification of GCC mRNA	qRT-PCR	[221]
